# Supplementary material for: Water immunity overrides stomatal immunity in plant resistance to Pseudomonas syringae
Source: Plant Physiol. 2025 Apr 2;198(1):kiaf127. doi: 10.1093/plphys/kiaf127 (PMC12063527; doi:10.1093/plphys/kiaf127)
Supplement: kiaf127_Supplementary_Data [file kiaf127_supplementary_data.pdf]

DOI	Reference	Title	Mutant lines	Bacterial strain	Result	Method	Treatment time	Photoperiod	Light intensity	Temperature (day/night)	Humidity (%)	Plant age
https://doi.org/10.1016/j.cell.2006.06.054.	Melotto et al., 2006	Plant Stomata Function in Innate Immunity against Bacterial Invasion	*ost1-2*, *aba3-1*	*Pst* DC3000, *Pst* DC3118	More bacterial growth observed in *ost1-2* vs. WT.	Vacuum infiltration and dipping	-	12h/12h	100 µE/m2/s	22°C	-	5-6 weeks
https://doi.org/10.1038/sj.emboj.7601575	de Torres-Zabala et al., 2007	Pseudomonas syringae pv. *tomato* hijacks the *Arabidopsis* abscisic acid signalling pathway to cause disease	*abi2-1*, *abi1-1*	*Pst* DC3000	Less bacterial growth in *abi* mutants vs. wildtype (20-80 times less)	Syringe infiltration	-	12h/12h	100–150 µEinsteins	22°C/20°C	70 %	5-6 weeks
https://doi.org/10.1371/journal.pbio.1000139	Liu et al., 2009	RIN4 Functions with Plasma Membrane H+-ATPases to Regulate Stomatal Apertures during Pathogen Attack	*ost2-2d*	*Pst* DC3000, *Pst* DC3000 (AvrRpt2), DC 000 *flaA−*	More bacterial growth in Ost2-2d vs. Col-0. The difference is bigger after syringe infiltration, and less distinctive after spray inoculation.	Spraying and syringe-infiltration	-	10h/14h	85 µE/m2/s	24°C	-	4-5 weeks
https://doi.org/10.1104/pp.110.157016	Zeng & He, 2010	A Prominent Role of the Flagellin Receptor FLAGELLIN-SENSING2 in Mediating Stomatal Response to *Pseudomonas syringae* pv *tomato* DC3000 in Arabidopsis	*fls2*, *efr1*, *fls2 efr1*	*Pst* DC3000, *Pst* DC3118	More bacterial growth in *fls2* and *fls2 efr1* mutants vs. Col-0	Dip-inoculation and syringe infiltration	-	16h/8h	80 to 100 µE m−2 s−1	20°C	-	5 weeks
https://doi.org/10.1111/j.1365-313X.2012.05116.x	Kumar et al., 2012	Rhizobacteria *Bacillus subtilis* restricts foliar pathogen entry through stomata	*ost1-1*	*Pst* DC3000	No significant differences in bacterial growth between Col-0 and *ost1-1*	Dipping rosettes and pressure infiltration	-	16h/8h	110 µE/m2/s	20°C	60 %	-
https://doi.org/10.1371/journal.pbio.1001513	Montillet et al., 2013	An Abscisic Acid-Independent Oxylipin Pathway Controls Stomatal Closure and Immune Defense in *Arabidopsis*	*lox1-1*, *lox1-2* for *Pst* growth	*Pst* DC3000	More bacterial growth in mutants vs. Col-0	Spray-inoculation	-	8h/16h	150 - 200 µE/m2/s	22°C	65 %	4 weeks
https://doi.org/10.1105/tpc.15.00466	Zhou et al., 2015	An Arabidopsis Plasma Membrane Proton ATPase Modulates JA Signaling and Is Exploited by the *Pseudomonas syringae* Effector Protein AvrB for Stomatal Invasion	*ost2-2D*, *ost2-2D coi1*	*Pst* DC3000, *Pst* cor-, Pst cor- avrB, *Pst* cor- avrBT125A, and *Pst* cor-avrBR266G	More bacterial growth after spray-inoculation in *ost2-2D* vs. wild type (5 times more), but same amount (or slightly less) when syringe-infiltrated	Spray-inoculation (3 days after spraying) and infiltration	-	10h/14h	-	23°C	-	4 weeks
https://doi.org/10.1104/pp.17.00220	Jalakas et al., 2017	The Role of ENHANCED RESPONSES TO ABA1 (ERA1) in Arabidopsis Stomatal Responses Is Beyond ABA Signaling	*ost1-3*	*Pst* DC3000, *Pst* DC3118	No significant differences	Surface inoculation (dipping).	-	9h/15h	~100 µE m−2 s−1	22-24°C / 17-19°C	-	5 weeks
https://doi.org/10.1080/19420889.2018.1495007	Kurusu et al., 2018	Involvement of S-type anion channels in disease resistance against an oomycete pathogen in Arabidopsis seedlings	*slac1-3* and *slah3-4*	*Pst* DC3000, *Pst* DC3000 (*avrRpt2*)	No significant difference in bacterial growth between *slac1*/*slah3* vs. wild type	Syringe infiltration	-	16 h /8 h	-	22°C	-	3 weeks
https://doi.org/10.1016/j.chom.2022.02.002	Hu et al., 2022	Bacterial effectors manipulate plant abscisic acid signaling for creation of an aqueous apoplast	*ost1/snrk2.6*, *slac1*	*Pst* DC3000, *avrE*, *hopM1*- and *avrE-/hopM1-*	Less bacteria in *ost1 / snrk2.6 and slac1* (Fig. S4D) after infiltration, 2 days post inoculation.	Syringe infiltration	-	12h/12h	-	22°C	65 %	4 weeks
https://doi.org/10.1016/j.jplph.2021.153585	Ou et al., 2022	Calcium-dependent ABA signaling functions in stomatal immunity by regulating rapid SA responses in guard cells	*ost1-3*	*Pst* DC3000, *Pst* DC3118	No significant differences in bacterial growth between Col-0 and *ost1-3*	Surface inoculation (dipping).	-	10 h/14 h	125 µmol m-2 s-1.	22°C	-	4-6 weeks
https://doi.org/10.1111/tpj.16380	Arnaud et al., 2023	RBOHF activates stomatal immunity by modulating both reactive oxygen species and apoplastic pH dynamics in Arabidopsis	*rbohD*, *rbohF*	*Pst* DC3000 COR−	More bacterial growth in *rbohF* vs. wild type and *rbohD*, both after syringe and spray inoculation.	Spraying and syringe-infiltration	5-6 hours after light switch was on	10 h/14 h	100 µmol m−2 sec−1	22°C/19°C	60 %	4-5 weeks

Supplementary Table S1. Literature describing discrepancies in mutant resistance to *Pst* and differing growth conditions.

**AGI code**	**Gene name**	**Protein description and function**	**Mutant line name (aliases)**	**Stock name/reference**	**Stomatal density**	**Steady-state transpiration/stomatal conductance**
AT4G20940	*GUARD CELL HYDROGEN PEROXIDE-RESISTANT 1* (***GHR1***)	A leucine-rich repeat receptor-like pseudokinase involved in SLAC1 activation. Required for, e.g., darkness, ABA, CO_2 and ROS-induced stomatal closure. (e.g., Hua et al., 2012; Sierla et al., 2018)	*ghr1-3*	GK_760C07	Wild type-like (Tulva et al., 2025)	Enhanced (Sierla et al., 2018)
AT1G12480	*SLOW ANION CHANNEL 1* (***SLAC1***)	An anion efflux channel required for stomatal closure in response to various signals. Mediates S-type anion currents for membrane depolarization in stomatal closure (e.g., Vahisalu et al., 2008;, Negi et al., 2008;, Hedrich & Geiger, 2017)	*slac1-3*	SALK_099139	Wild type-like (Jalakas et al., 2021)	Enhanced (Jalakas et al., 2021)
AT4G17970	*ALUMINUM-ACTIVATED MALATE TRANSPORTER 12* (***ALMT12***)	Malate-sensitive anion transporter involved in stomatal closure. Belongs to R-type anion channels (e.g., Sasaki et a., 2010; Meyer et al.; 2010).	*almt12-1* (*quac1-1*)	SM_3_38592	Wild type-like (Jalakas et al., 2021)	Wild type-like (Jalakas et al., 2021)
AT5G24030	*SLAC1 HOMOLOGUE 3* (*SLAH3*)	An S-type anion efflux channel and a homolog of SLAC1, involved in stomatal closure (e.g., Geiger et al., 2011; Zhang et al., 2016).	*slah3-1*	GK-371G03	Not known	Wild type-like (Jalakas et al., 2021)
			almt12-1 slac1-3 slah3-1 (triple mutant)	Jalakas et al., 2021	Wild type-like (Jalakas et al., 2021)	Enhanced (Jalakas et al., 2021)
AT4G33950	*OPEN STOMATA 1* (*OST1*)	Central kinase required for ABA-mediated stomatal closure. Phosphorylates various targets including SLAC1 (e.g., Mustilli et al., 2002; Belin et al., 2006; Merilo et al., 2013)	*ost1-3* (*srk2e*)	SALK_008068	Wild type-like (Jalakas et al., 2018)	Enhanced (Jalakas et al., 2018)
AT2G18960	*H(+)-ATPASE 1* (***AHA1***) / *OPEN STOMATA 2* (*OST2*)	Plasma membrane H+ ATPase. Increases guard cell membrane polarisation via proton transport, promoting stomatal opening when active. Functions e.g., in blue light-mediated stomatal opening and plant development (e.g., Ueno et al., 2005; Merlot et al., 2007; Yamauchi et al., 2016).	*ost2-2d*	Merlot et al., 2007	Wild type-like (Costa et al., 2015)	Enhanced (Costa et al., 2015)
AT2G42620	*MORE AXILLARY GROWTH 2* (***MAX2***)	A component of SCF (ASK-cullin-F-box) E3 ubiquitin ligase complexes, functioning in ubiquitination and proteasomal degradation of proteins. Involved in strigolactone signalling, and regulates plant immunity, axillary shoot repression and branching (e.g., Stirnberg et al., 2002; Piisilä et al., 2015; Kalliola et al., 2020).	*max2-4*	SALK_028336	Wild type-like in *max2-1* (Bu et al., 2014)	Enhanced (Kalliola et al., 2020)
AT1G62400	*HIGH LEAF TEMPERATURE 1* (***HT1***)	A kinase involved in CO_2-sensing and stomatal control via MPK12/4 interaction. Regulates CO_2-induced stomatal movements (e.g., Hashimoto et al., 2006; Hõrak et al., 2016; Takahashi et al., 2022; Yeh et al., 2023).	*ht1-2*	Hashimoto et al., 2006	Wild type-like (Hashimoto et al., 2006)	Decreased (Hashimoto-Sugimoto et al., 2016)

Supplementary Table S2. Mutant lines used in this study with their gene function and known stomatal phenotypes.

**Mutant line name (aliases)**	**Forward primer**	**Reverse primer**	**T-DNA specific primer/CAPS enzyme**
ghr1-3	CTCCTTATCTGGACCTTTGCC	TGGCCCTTCTAAGCTGTTAGA	ATATTGACCATCATACTCATTGC (GABI-insert pAC161)
slac1-3	AACTTCTTCTTCGCTCCTTGG	GACCATTTCTTTGCCTGTTTG	GCGTGGACCGCTTGCTGCAACT (SALK Lbb1)
almt12-1 (*quac1-1*)	GTTGTGCAAAGGGCTTAATAGAG	CAAGAAGGCTCATGAAAAGACAG	TACGAATAAGAGCGTCCATTTTAGAGT (Spm32)
slah3-1	ACCCCATTTCCACCTTCGGTATG	GGATAATGGTGGTCACGAGCAG	ATATTGACCATCATACTCATTGC (GABI-insert pAC161)
almt12-1 slac1-3 slah3-1 (triple mutant)			
ost1-3 (*srk2e*)	CATATCTTTAGACGAGGGGCC	GTGAGTGGTCCAATGGATTTG	GCGTGGACCGCTTGCTGCAACT (SALK Lbb1)
ost2-2d	GGAGTGAACAAGAGGCTGCT	TTGGTCACAGGAAGGGACTC	CAPS (Hpy188III)
max2-4	GCTCTCACCTCACTATCCGTG	CATCCTCTCCCTATAGCCACC	GCGTGGACCGCTTGCTGCAACT (SALK Lbb1)
ht1-2	AGGATCCCAACACACAAGGA	CATCTCGTCGTTCAAAAGCA	CAPS (PsuI)

Supplementary Table S3. Primers for genotyping mutants by PCR.

**AGI code**	**Gene**	**Forward primer**	**Reverse primer**	**Primer efficiency**	
At1g74710	*ICS1*	GCTTGGCTAGCACAGTTACAGC	CACTGCAGACACCTAATTGAGTCC	1.928	
At1g19180	*JAZ1*	CGTGGCTCGGTTTAGCAG	TGAAGCAACGTCGTCAAAAG	1.801	
At1g72520	*LOX4*	CTAGCCGTAGGAATCGCTGT	TACACGTAACACCCGGTTCA	1.85	
At3G14440	*NCED3*	TCGAAGCAGGGATGGTCAACAG	GCTCGGCTAAAGCCAAGTAAGC	1.752	
At2G39800	*P5CS1*	AGAGGATCACGAAGTTGCAGAGC	TGTGGAACACAGCAGCGCTATC	1.867	
					M-value
At4g34270	*TIP41*	GTGAAAACTGTTGGAGAGAAGCAA	TCAACTGGATACCCTTTCGCA	1.867	0.088-0.148
At5g08290	*YLS8*	TTACTGTTTCGGTTGTTCTCCATTT	CACTGAATCATGTTCGAAGCAAGT	1.822	0.07-0.182
At1g13320	*PP2AA3*	GCGGTTGTGGAGAACATGATACG	GAACCAAACACAATTCGTTGCTG	1.854	0.075-0.136

Supplementary Table S4. RT-qPCR primers and expression stability of reference genes.

References

Arnaud, D., Deeks, M.J. and Smirnoff, N. (2023). RBOHF activates stomatal immunity by modulating both reactive oxygen species and apoplastic pH dynamics in Arabidopsis. *The Plant Journal*, 116(2), pp.404–415. doi:https://doi.org/10.1111/tpj.16380

Belin, C., de Franco, P.-O., Bourbousse, C., Chaignepain S., Schmitter, J.-M., Vavasseur, A., Giraudat J., Barbier-Brygoo H. and Thomine S. (2006). Identification of Features Regulating OST1 Kinase Activity and OST1 Function in Guard Cells . *Plant Physiology*, 141(4), pp.1316–1327. doi:https://doi.org/10.1104/pp.106.079327.

Bu, Q., Lv, T., Shen, H., Luong, P., Wang, J., Wang, Z., Huang, Z., Xiao, L., Engineer, C., Kim, T.H., Schroeder, J.I. and Huq, E. (2013). Regulation of Drought Tolerance by the F-Box Protein MAX2 in Arabidopsis. *Plant Physiology*, 164(1), pp.424–439. doi:https://doi.org/10.1104/pp.113.226837.

Costa, J.M., Monnet, F., Jannaud, D., Leonhardt, N., Ksas, B., Reiter, I.M., Pantin, F. and Genty, B. (2015). OPEN ALL NIGHT LONG: The Dark Side of Stomatal Control. *Plant Physiology*, 167(2), pp.289–294. doi:https://doi.org/10.1104/pp.114.253369.

de Torres-Zabala, M., Truman, W., Bennett, M.H., Lafforgue, G., Mansfield, J.W., Rodriguez Egea, P., Bögre, L. and Grant, M. (2007). Pseudomonas syringae pv. tomato hijacks the Arabidopsis abscisic acid signalling pathway to cause disease. *The EMBO Journal*, 26(5), pp.1434–1443. doi:https://doi.org/10.1038/sj.emboj.7601575

Geiger, D., Maierhofer, T., AL-Rasheid, K.A.S., Scherzer, S., Mumm, P., Liese, A., Ache, P., Wellmann, C., Marten, I., Grill, E., Romeis, T. and Hedrich, R. (2011). Stomatal Closure by Fast Abscisic Acid Signaling Is Mediated by the Guard Cell Anion Channel SLAH3 and the Receptor RCAR1. *Science Signaling*, 4(173), pp.ra32–ra32. doi:https://doi.org/10.1126/scisignal.2001346.

Hashimoto, M., Negi, J., Young, J., Israelsson, M., Schroeder, J.I. and Iba, K. (2006). Arabidopsis HT1 kinase controls stomatal movements in response to CO2. *Nature Cell Biology*, 8(4), pp.391–397. doi:https://doi.org/10.1038/ncb1387.

Hashimoto-Sugimoto, M., Negi, J., Monda, K., Higaki, T., Yasuhiro Isogai, Nakano, T., Seiichiro Hasezawa and Iba, K. (2016). Dominant and recessive mutations in the Raf-like kinaseHT1gene completely disrupt stomatal responses to CO2in Arabidopsis. *Journal of Experimental Botany*, 67(11), pp.3251–3261. doi:https://doi.org/10.1093/jxb/erw134.

Hedrich, R. and Geiger, D. (2017). Biology of SLAC1-type anion channels - from nutrient uptake to stomatal closure. *New Phytologist*, 216(1), pp.46–61. doi:https://doi.org/10.1111/nph.14685.

Hõrak, H., Sierla, M., Tõldsepp, K., Wang, C., Wang, Y.-S., Nuhkat, M., Valk, E., Pechter, P., Merilo, E., Salojärvi, J., Overmyer, K., Loog, M., Brosché, M., Schroeder, J.I., Kangasjärvi, J. and Kollist, H. (2016). A Dominant Mutation in the HT1 Kinase Uncovers Roles of MAP Kinases and GHR1 in CO2-Induced Stomatal Closure. *The Plant Cell*, 28(10), pp.2493–2509. doi:https://doi.org/10.1105/tpc.16.00131.

Hu, Y., Ding, Y., Cai, B., Qin, X., Wu, J., Yuan, M., Wan, S., Zhao, Y. and Xin, X.-F. (2022). Bacterial effectors manipulate plant abscisic acid signaling for creation of an aqueous apoplast. *Cell Host & Microbe*, 30(4), pp.518-529.e6. doi:https://doi.org/10.1016/j.chom.2022.02.002

Hua, D., Wang, C., He, J., Liao, H., Duan, Y., Zhu, Z., Guo, Y., Chen, Z. and Gong, Z. (2012). A Plasma Membrane Receptor Kinase, GHR1, Mediates Abscisic Acid- and Hydrogen Peroxide-Regulated Stomatal Movement in Arabidopsis. *The Plant Cell*, 24(6), pp.2546–2561. doi:https://doi.org/10.1105/tpc.112.100107.

Jalakas, P., Huang, Y.-C., Yeh, Y.-H., Zimmerli, L., Merilo, E., Kollist, H. and Brosché, M. (2017). The Role of ENHANCED RESPONSES TO ABA1 (ERA1) in Arabidopsis Stomatal Responses Is Beyond ABA Signaling. *Plant Physiology,* 174(2), pp.665–671. doi:https://doi.org/10.1104/pp.17.00220

Jalakas, P., Merilo, E., Kollist, H., & Brosché, M. (2018). ABA-mediated regulation of stomatal density is OST1-independent. *Plant Direct*, 2(9), e00082. doi:https://doi.org/10.1002/pld3.82

Jalakas, P., Nuhkat, M., Vahisalu, T., Merilo, E., Brosché, M. and Kollist, H. (2021). Combined action of guard cell plasma membrane rapid- and slow-type anion channels in stomatal regulation. *Plant Physiology*, 187(4), pp.2126–2133. doi:https://doi.org/10.1093/plphys/kiab202.

Kalliola, M., Jakobson, L., Davidsson, P., Pennanen, V., Waszczak, C., Yarmolinsky, D., Zamora, O., Palva, E.T., Kariola, T., Kollist, H. and Brosché, M. (2020). Differential role of MAX2 and strigolactones in pathogen, ozone, and stomatal responses. *Plant Direct*, 4(2). doi:https://doi.org/10.1002/pld3.206.

Kumar, A.S., Lakshmanan, V., Caplan, J.L., Powell, D., Czymmek, K.J., Levia, D.F. and Bais, H.P. (2012). Rhizobacteria Bacillus subtilisrestricts foliar pathogen entry through stomata. *The Plant Journal*, 72(4), pp.694–706. doi:https://doi.org/10.1111/j.1365-313X.2012.05116.x

Kurusu, T., Mitsuka, D., Yagi, C., Kitahata, N., Tsutsui, T., Ueda, T., Yamamoto, Y., Negi, J., Iba, K., Betsuyaku, S., and Kuchitsu, K. (2018). Involvement of S-type anion channels in disease resistance against an oomycete pathogen in Arabidopsis seedlings. *Communicative & Integrative Biology*, 11(3), pp.1–6. doi:https://doi.org/10.1080/19420889.2018.1495007.

Liu, J., Elmore, J. M., Fuglsang, A. T., Palmgren, M. G., Staskawicz, B. J., & Coaker, G. (2009). RIN4 functions with plasma membrane H+-ATPases to regulate stomatal apertures during pathogen attack. *PLoS biology*, 7(6), e1000139. doi:https://doi.org/10.1371/journal.pbio.1000139

Melotto, M., Underwood, W., Koczan, J., Nomura, K. and He, S.Y. (2006). Plant Stomata Function in Innate Immunity against Bacterial Invasion. *Cell*, 126(5), pp.969–980. doi:https://doi.org/10.1016/j.cell.2006.06.054.

Merilo, E., Laanemets, K., Hu, H., Xue, S., Jakobson, L., Tulva, I., Gonzalez-Guzman, M., Rodriguez, P.L., Schroeder, J.I., Brosche, M. and Kollist, H. (2013). PYR/RCAR Receptors Contribute to Ozone-, Reduced Air Humidity-, Darkness-, and CO2-Induced Stomatal Regulation. *Plant Physiology*, 162(3), pp.1652–1668. doi:https://doi.org/10.1104/pp.113.220608.

Merlot, S., Leonhardt, N., Fenzi, F., Valon, C., Costa, M., Piette, L., Vavasseur, A., Genty, B., Boivin, K., Müller, A., Giraudat, J. and Leung, J. (2007). Constitutive activation of a plasma membrane H+-ATPase prevents abscisic acid-mediated stomatal closure. *The EMBO Journal*, 26(13), pp.3216–3226. doi:https://doi.org/10.1038/sj.emboj.7601750.

Meyer, S., Mumm, P., Imes, D., Endler, A., Weder, B., Al-Rasheid, K.A.S., Geiger, D., Marten, I., Martinoia, E. and Hedrich, R. (2010). AtALMT12 represents an R-type anion channel required for stomatal movement in Arabidopsis guard cells. *The Plant Journal*, 63(6), pp.1054–1062. doi:https://doi.org/10.1111/j.1365-313x.2010.04302.x.

Montillet, J.-L., Leonhardt, N., Mondy, S., Tranchimand, S., Rumeau, D., Boudsocq, M., Garcia, A.V., Douki, T., Bigeard, J., Laurière, C., Chevalier, A., Castresana, C. and Hirt, H. (2013). An Abscisic Acid-Independent Oxylipin Pathway Controls Stomatal Closure and Immune Defense in Arabidopsis. *PLoS Biology*, 11(3), p.e1001513. doi:https://doi.org/10.1371/journal.pbio.1001513

Mustilli, A. C., Merlot, S., Vavasseur, A., Fenzi, F., & Giraudat, J. (2002). Arabidopsis OST1 protein kinase mediates the regulation of stomatal aperture by abscisic acid and acts upstream of reactive oxygen species production. *The Plant Cell*, *14*(12), 3089-3099. doi:https://doi.org/10.1105/tpc.007906

Negi, J., Matsuda, O., Nagasawa, T., Oba, Y., Takahashi, H., Kawai-Yamada, M., Uchimiya, H., Hashimoto, M. and Iba, K. (2008). CO2 regulator SLAC1 and its homologues are essential for anion homeostasis in plant cells. *Nature*, 452(7186), pp.483–486. doi:https://doi.org/10.1038/nature06720.

Ou, X., Li, T., Zhao, Y., Chang, Y., Wu, L., Chen, G., Day, B. and Jiang, K. (2022). Calcium-dependent ABA signaling functions in stomatal immunity by regulating rapid SA responses in guard cells. *Journal of Plant Physiology*, 268, p.153585. doi:https://doi.org/10.1016/j.jplph.2021.153585

Piisilä, M., Keceli, M.A., Brader, G., Jakobson, L., Jõesaar, I., Sipari, N., Kollist, H., Palva, E. and Kariola, T. (2015). The F-box protein MAX2 contributes to resistance to bacterial phytopathogens in Arabidopsis thaliana. *BMC Plant Biology*, 15(1), p.53. doi:https://doi.org/10.1186/s12870-015-0434-4.

Sasaki, T., Mori, I.C., Teiichi Furuichi, Shintaro Munemasa, Toyooka, K., Matsuoka, K., Murata, Y. and Yamamoto, Y. (2010). Closing Plant Stomata Requires a Homolog of an Aluminum-Activated Malate Transporter. *Plant and Cell Physiology*, 51(3), pp.354–365. doi:https://doi.org/10.1093/pcp/pcq016.

Sierla, M., Hõrak, H., Overmyer, K., Waszczak, C., Yarmolinsky, D., Maierhofer, T., Vainonen, J.P., Salojärvi, J., Denessiouk, K., Laanemets, K., Tõldsepp, K., Vahisalu, T., Gauthier, A., Puukko, T., Paulin, L., Auvinen, P., Geiger, D., Hedrich, R., Kollist, H. and Kangasjärvi, J. (2018). The Receptor-like Pseudokinase GHR1 Is Required for Stomatal Closure. *The Plant Cell*, 30(11), pp.2813–2837. doi:https://doi.org/10.1105/tpc.18.00441.

Stirnberg, P., van de Sande, K. and Leyser, H.M.O. (2002). MAX1 and MAX2 control shoot lateral branching in Arabidopsis. *Development*, 129(5), pp.1131–1141. doi:https://doi.org/10.1242/dev.129.5.1131.

Tulva, I., Jalakas, P., Ivandi, E., Wierzchula, A., Obinwanne, P.K. and Horak, H. (2025). Low relative air humidity leads to smaller, denser stomata and higher stomatal ratios in Arabidopsis. *bioRxiv* doi:https://doi.org/10.1101/2025.01.31.635895.

Ueno, K., Kinoshita, T., Inoue, S., Emi, T. and Shimazaki, K. (2005). Biochemical Characterization of Plasma Membrane H+-ATPase Activation in Guard Cell Protoplasts of Arabidopsis thaliana in Response to Blue Light. *Plant and Cell Physiology*, 46(6), pp.955–963. doi:https://doi.org/10.1093/pcp/pci104.

Vahisalu, T., Kollist, H., Wang, Y.-F., Nishimura, N., Chan, W.-Y., Valerio, G., Lamminmäki, A., Brosché, M., Moldau, H., Desikan, R., Schroeder, J.I. and Kangasjärvi, J. (2008). SLAC1 is required for plant guard cell S-type anion channel function in stomatal signalling. *Nature*, 452(7186), pp.487–491. doi:https://doi.org/10.1038/nature06608.

Wang, Y.-F., Zhang, A., Ren, H.-M., Tan, Y.-Q., Qi, G.-N., Yao, F.-Y., Wu, G.-L., Yang, L.-W., Hussain, J. and Sun, S.-J. (2016). S-type Anion Channels SLAC1 and SLAH3 Function as Essential Negative Regulators of Inward K+ Channels and Stomatal Opening in Arabidopsis. *The Plant Cell*, p.tpc.01050.2016. doi:https://doi.org/10.1105/tpc.16.01050.

Yamauchi, S., Takemiya, A., Sakamoto, T., Kurata, T., Tsutsumi, T., Kinoshita, T. and Shimazaki, K. (2016). The Plasma Membrane H+-ATPase AHA1 Plays a Major Role in Stomatal Opening in Response to Blue Light. *Plant Physiology*, 171(4), pp.2731–2743. doi:https://doi.org/10.1104/pp.16.01581.

Yeh, C.-Y., Wang, Y.-S., Takahashi, Y., Katarina Kuusk, Paul, K., Triinu Arjus, Oleksii Yadlos, Schroeder, J.I., Ivar Ilves, García-Sosa, A.T. and Hannes Kollist (2023). MPK12 in stomatal CO_2 signaling: function beyond its kinase activity. *New Phytologist*, 239(1), pp.146–158. doi:https://doi.org/10.1111/nph.18913.

Zeng, W. and He, S.Y. (2010). A Prominent Role of the Flagellin Receptor FLAGELLIN-SENSING2 in Mediating Stomatal Response to Pseudomonas syringae pv tomato DC3000 in Arabidopsis. *Plant Physiology*, 153(3), pp.1188–1198. doi:https://doi.org/10.1104/pp.110.157016.

Zhou, Z., Wu, Y., Yang, Y., Du, M., Zhang, X., Guo, Y., Li, C. and Zhou, J. (2015). An Arabidopsis Plasma Membrane Proton ATPase Modulates JA Signaling and Is Exploited by the Pseudomonas syringae Effector Protein AvrB for Stomatal Invasion. *The Plant Cell*, 27(7), 2032-2041. doi:https://doi.org/10.1105/tpc.15.00466
